# Effects of Transcranial Direct Current Stimulation on Hand Dexterity in Multiple Sclerosis: A Design for a Randomized Controlled Trial

**DOI:** 10.3390/brainsci10030185

**Published:** 2020-03-23

**Authors:** Samar S. Ayache, Naji Riachi, Rechdi Ahdab, Moussa A. Chalah

**Affiliations:** 1 EA 4391, Excitabilité Nerveuse et Thérapeutique, Université Paris-Est-Créteil, 94010 Créteil, France ; samar.ayache@aphp.fr (S.S.A.); moussa.chalah@u-pec.fr (M.A.C.); 2 Service de Physiologie – Explorations Fonctionnelles, Hôpital Henri Mondor, Assistance Publique–Hôpitaux de Paris, 94010 Créteil, France; 3 Neurology Division, Lebanese American University Medical Center Rizk Hospital, Beirut 113288, Lebanon; rechdi.ahdab@laumcrh.com; 4 Gilbert and Rose Mary Chagoury School of Medicine School of Medicine, Lebanese American University, Byblos 4504, Lebanon

**Keywords:** multiple sclerosis, transcranial direct current stimulation, tDCS, motor cortex, cerebellum, hand dexterity

## Abstract

Background: Cerebellar and motor tracts are frequently impaired in multiple sclerosis (MS). Altered hand dexterity constitutes a challenge in clinical practice, since medical treatment shows very limited benefits in this domain. Cerebellar control is made via several cerebellocortical pathways, of which the most studied one links the cerebellum to the contralateral motor cortex via the contralateral ventro-intermediate nucleus of the thalamus influencing the corticospinal outputs. Modulating the activity of the cerebellum or of the motor cortex could be of help. Method: The main interest here is to evaluate the efficacy of transcranial direct current stimulation (tDCS), a noninvasive brain stimulation technique, in treating altered dexterity in MS. Forty-eight patients will be recruited in a randomized, double-blind, sham-controlled, and crossover study. They will randomly undergo one of the three interventions: anodal tDCS over the primary motor area, cathodal tDCS over the cerebellum, or sham. Each block consists of five consecutive daily sessions with direct current (2 mA), lasting 20 min each. The primary outcome will be the improvement in manual dexterity according to the change in the time required to complete the nine-hole pegboard task. Secondary outcomes will include fatigue, pain, spasticity, and mood. Patients’ safety and satisfaction will be rated. Discussion: Due to its cost-effective, safe, and easy-to-use profile, motor or cerebellar tDCS may constitute a potential tool that might improve dexterity in MS patients and therefore ameliorate their quality of life.

## 1. Introduction

Multiple sclerosis (MS) is a chronic progressive inflammatory disease of the central nervous system and represents a major cause of non-traumatic disability in young adults [1,2]. Throughout the disease course, patients experience sensorimotor, emotional, cognitive, and behavioral symptoms. Among these symptoms, hand movements are frequently altered in MS; this results in a significant amount of disability [3,4,5].

Hand function is crucial for everyday life and its alteration, like in the context of MS, might constitute a real obstacle to living an independent life. In normal individuals, hand movements are perfectly balanced and coordinated allowing to execute functions with marvelous precision. “Dexterity” refers to the precise manipulation of objects and plays an important role in daily activities [6,7]. Performance of any activity requires complex sensory feedback from the external environment, sensorimotor integration, motor planning, execution, and adaptation. Such interaction plays an important role in adapting movement to environmental changes through rapid adjustments in motor control [8,9,10]. Data from neuroimaging studies have shown the contribution of many cerebral cortical areas (i.e., of motor cortex, cerebellum, and basal ganglia) in providing the optimal dexterity [11,12,13,14,15,16,17,18,19]. Motor cortex and basal ganglia generate voluntary and automatic movements, respectively. The cerebellum receives and integrates multisensory inputs (proprioceptive, visual, tactile, and vestibular), and adjusts the motor output accordingly [20]. Therefore, the cerebellum contributes to various aspects of motor control, such as postural stabilization, coordination, precision, and timing of movements. Cerebellar control is made via several pathways; the most studied one being the cerebellothalamocortical pathway. The latter links the cerebellum with the contralateral motor cortex via the contralateral ventro-intermediate nucleus of the thalamus and therefore could influence the corticospinal outputs [21,22,23]. The available literature supports the existence of a tonic facilitation of the motor activity exerted by the deep cerebellar nuclei, whereas putting the Purkinje cells in action would result in phasic inhibition of the mentioned nuclei and therefore “disfacilitation” of motor circuits [24].

In case of cerebellar dysfunction resulting from MS-related pathological changes, performing precise movement might be impossible. In fact, altered hand dexterity could affect 76% of MS patients, is associated with the deterioration of the overall upper limb activity and disuse, increases the need for more assistance and the perceived difficulties with activities of daily living [25,26,27]. Facing this source of disability, the available medical treatments showed very limited benefits in improving hand dexterity. Noninvasive brain stimulation (NIBS) techniques seem appealing tools to treat various neuropsychiatric complaints, including those encountered in patients with MS [28]. Transcranial direct current stimulation (tDCS) is a relatively new NIBS technique with a good safety profile, easy implementation, good patients’ tolerance, and little or no adverse effects [29,30,31,32,33,34,35]. It could modify spontaneous neuronal excitability and activity by tonic depolarization (anodal tDCS) or hyperpolarization (cathodal tDCS) of the neuronal resting membrane potential [36,37]. To date, tDCS effects on hand dexterity have not been explored in MS [38]. The main objective of this work is to evaluate the effects in question. Stimulating the motor cortex, or removing the cerebellar inhibition over it, can be beneficial in improving dexterity in MS patients. Therefore, in this work, we thought of activating (anodal tDCS) the primary motor cortex (M1) contralateral to the most affected hand or inhibiting (cathodal tDCS) the cerebellar cortex (i.e., Purkinje cells) aiming to facilitate motor output.

## 2. Materials and Methods

This is a randomized, double-blind, crossover, and sham-controlled study. A crossover design has been chosen, so that each participant can act as his or her own control in an attempt to limit the inter-subject variability. In addition, such a design requires a smaller sample size compared to a parallel group design for the same statistical power [39]. However, one should acknowledge the limitations of this within-subject design, mainly, the time burden imposed on patients (longer participation time compared to the parallel-group design), and the possibility of a carry-over effect (e.g., potential neuroplastic effects induced by the preceding tDCS effects) or an order effect. The former effect cannot be formally ruled out, but was taken into consideration by including a washout period of 3 weeks between tDCS blocks, as done previously [40]. The latter effect could be controlled by counterbalancing the tDCS order among patients and could be statistically tested by adding the order as a covariate in the analysis of covariance (ANCOVA).

The study was approved by the institutional review board of the Lebanese American University, Lebanon (IRB registration #IRB00006954). All patients will voluntarily give their written informed consent prior to inclusion. The study will take place in conformity with the Declaration of Helsinki and its amendments.

### 2.1. Study Participants

Patients presenting for consultation or hospitalization to the Neurology Department will be screened for eligibility. Patients (men and women) will be included if they fulfill the following criteria: (i) age between 18 and 70 years, (ii) presence of unilateral or bilateral upper extremity clumsiness as assessed by Chedoke Arm and Hand Activity Inventory version 13 (CAHAI-13) [41], (iii) definite MS diagnosis (relapsing-remitting, primary progressive, or secondary progressive) according to the 2017 McDonald criteria [42], and (iv) stable pharmacological and physical treatment for at least one month. Patients will be excluded if they have (i) had a relapse within the previous two months, (ii) severe upper limb weakness according to the Medical Research Council (MRC) grading system (MRC < 3 of the arm, forearm, or hand) [43], (iii) severe upper limbs spasticity as per the Modified Ashworth scale (MAS > 2) [44], (iv) severe dysdiadochokinesia (> 2) or dysmetria (>2) according to the ataxia scale [45], (v) severe sensory impairment as per the Semmes–Weinstein monofilament test (absence of sensation using monofilament E) [46], (vi) severe physical disability as per the Expanded Disability Status Scale (EDSS) (≥6.5) [47], (vii) apraxia as per the Apraxia Screen of TULIA (AST < 9) [48], (vii) depression as per the Beck Depression Inventory (BDI > 19) [49], or (viii) other neurologic and psychiatric diseases.

### 2.2. tDCS

**Figure 1:**
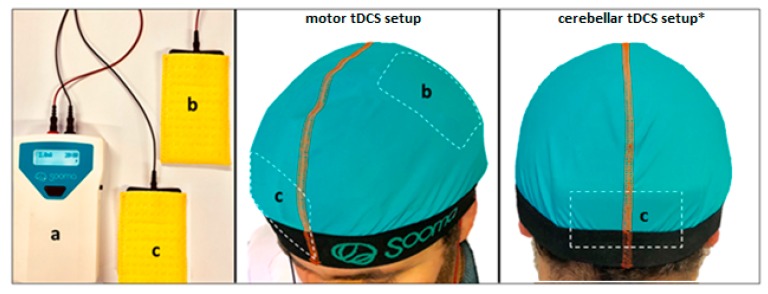
An illustration of a tDCS setup (Sooma Oy, Helsinki, Finland). a. Battery-driven stimulator. b. Anode. c. Cathode. * The reference electrode over the shoulder is not shown.

A battery-driven stimulator (Sooma Oy, Helsinki, Finland) will deliver the current at an intensity of 2 mA through sponge electrodes soaked in a saline solution and having a surface area of 35 cm^2^ (Figure 1). For the real tDCS treatment, the current will be ramped up for 15 s until reaching 2 mA which will be maintained for 20 min; then it will be ramped down for 15 s at the end of stimulation. The current intensity, stimulation duration, and number of sessions were adopted from previous studies done on MS and other clinical populations [50,51]. For the sham condition, the current will be ramped up for 15 s, then progressively phased off, providing effective blinding [52,53]. The anode and the cathode will be placed according to the following design: for anodal motor tDCS (real/sham), the anode will be centered over M1 (at C3/C4, according to the international 10-20 EEG system) contralaterally to the most affected side with its reference electrode (cathode) placed over the contralateral forehead (AF8, 10-20 EEG system) (Figure 1). For cathodal cerebellar tDCS (real/sham), the cathode will be centered on the median line 2 cm below the inion with its lateral borders about 1 cm medially to the mastoid apophysis; its reference electrode (anode) will be placed over the right shoulder based on previous cerebellar tDCS studies (Figure 1) [54,55,56,57,58]. Preliminary modeling studies demonstrated that in this setup, the electric field distribution generated by cerebellar tDCS symmetrically involved the cerebellum, where the electric field distribution attained its peak.

### 2.3. Sample Size, Randomization and Blinding Effectiveness

In the absence of published data on this topic, the statistical outcomes of this study can serve in understanding and guiding the therapies of altered dexterity in MS patients. Previous tDCS studies addressing dexterity in stroke patients adopted sample sizes between 10 and 19 and yielded statistical significance [59,60].

In the absence of sham-controlled studies on this matter, sample size calculation was performed using the G*Power Software (version 3.1.9.6 [61]). A 2 × 3 within-subjects repeated measures analysis of variance was considered, with the factor time (before and after each stimulation condition) and the factor stimulation condition (active cathodal cerebellar, active anodal motor vs. sham). Considering the predicted medium effect size (f = 0.25) and adopting a two-tailed significant difference of α = 0.05 and an estimated power of 80%, the sample size estimate is 36. Taking into consideration a dropout rate of up to 25%, a sample of 45 patients will be sufficient to observe significant effects. *N* = 48 was adopted to be able to counterbalance the order of conditions as will be explained below.

**Figure 2:**
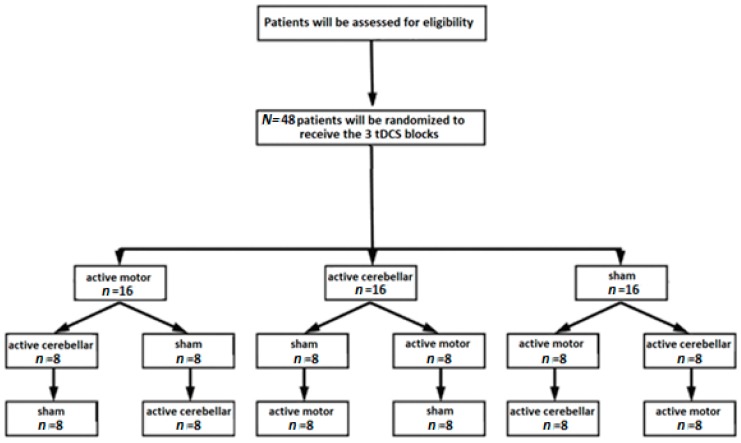
Flow chart of the study protocol.

Randomization will be performed by a researcher who will not be in contact with patients. In the first step, six different sets of blocks will be assigned, and each contains four patients: (active cathodal cerebellar, sham, active anodal motor), set B (active cathodal cerebellar, active anodal motor, sham), set C (sham, active cathodal cerebellar, active anodal motor), set D (sham, active anodal motor, active cathodal cerebellar), set E (active anodal motor, sham, active cathodal cerebellar), and set F (active anodal motor, active cathodal cerebellar, sham). In the second step, each set will be split into two subsets according to the sham setup, so that two patients per set will receive sham over the motor cortex (setup similar to active condition), and the other ones will receive sham over the cerebellum (setup similar to active condition). The flow chart of the protocol is displayed in Figure 2. The evaluators will assess the patients before and after tDCS blocks and will be blind to the stimulation conditions. Patients will be blind to the stimulation condition.

### 2.4. Description of the Clinical Evaluation

#### 2.4.1. Primary outcome: hand dexterity

The Nine-Hole Peg Test (9-HPT) will be administered by the evaluating physician to assess hand dexterity [5,62,63]. Briefly, patients will be trained to perform the test for five consecutive trials. The inter-trial interval will be 1 min. For each trial, the subject begins with the hand resting beside the pegboard. The experimenter initiates the trials with a verbal ready–steady–go command, and times the trial with a digital stopwatch. After training trials, another five trials will be done, recorded, and their sum will be calculated.

The dependent variable is the number of pegs per second computed based on nine pegs placed compared to the time needed to finish the test [63]. In case of inability to complete the test, the variable corresponds to the number of pegs placed divided by the time limit of the test (300 s).

#### 2.4.2. Secondary outcomes

Fatigue will be assessed using the 21-item Modified Fatigue Impact Scale (MFIS), which is relatively short, robust, and reliable [64]. It contains three subscales: cognitive (10 items), physical (9 items), and psychosocial fatigue (2 items). The items are scored from 0 to 4 (0 = Never, 1 = Rarely, 2 = Sometimes, 3 = Often, 4 = Almost always), and respondents are asked to consider their experiences with fatigue during the past four weeks.

Pain will be evaluated using a 0-10 visual analog scale (VAS) for pain. Anxiety and depressive symptoms will be assessed using the Hospital Anxiety and Depression Scale (HADS) [65]. Spasticity, motor power, and cerebellar function will be evaluated using the MAS [44], MRC [43], and ataxia scale scores [45], respectively. The MAS grading will be performed for the three upper limb segments (shoulder adductor, elbow flexor, wrist flexor), obtaining scores that can range from 0 (no increase in muscle tone) to 4 (affected parts rigid in flexion or extension) [44]. The Clinical Global Impression (CGI) and the Comfort Rating Questionnaire (CRQ) will be used to assess patients’ clinical impression and potential side effects [35]. The CRQ evaluates possible side effects (pain perception, tingling, burning, fatigue, nervousness, disturbed concentration, disturbed visual perception, and headache) observed before, during, and 24 hours after stimulation on a 10-point Likert scale. In addition, it contains two dichotomous questions that assess sleep disturbance and phosphene perception during/after stimulation. At the end of the last session of each stimulation block, patients will be asked to guess the type of stimulation to assess for effective blinding (active/sham).

### 2.5. Experimental Protocol

The first visit is an inclusion visit, where the examining physician will perform the neurologic exam, and verify the inclusion and exclusion criteria. Patients will obtain information regarding the protocol, consent form, and the other questionnaires to be filled. They will be called in the following days to check if he/she agrees to participate in the study and therefore to plan the stimulation sessions. In case of acceptance, an appointment will be scheduled in order to sign the consent form and deliver a booklet containing the VAS for pain to be filled for one week prior to the stimulation week and during the week of stimulation. If present, medications and physical therapy will be kept the same as the previous month. Patients will be asked to restrict stimulant consumptions (e.g., caffeine, nicotine) at least three hours prior to their appointments.

Each patient will be randomly assigned to receive 3 blocks separated by a washout interval of three weeks (anodal tDCS over M1 contralateral to the most affected hand, cathodal tDCS over the cerebellum, and sham over either cortical site). Each block will consist of five 20-min stimulation sessions, applied on day 1 to day 5 (D1 to D5). On D1, dexterity will be quantified based on the time required to perform the 9-HPT. Each patient will be evaluated for fatigue (MFIS), anxiety and depression (HADS), spasticity (MAS), motor power (MRC), and cerebellar function (ataxia scale). Afterwards, tDCS will be performed in a quiet and well-illuminated room with the patient sitting in an armchair without any concurrent cognitive or motor task. The stimulation session will be repeated from D1 to D5. At the end of D5 session, each patient will undergo the same evaluation as done on D1 (9-HPT, MFIS, HADS, MAS, MRC, ataxia scale), as well as the CRQ, CGI, and will be delivered a VAS booklet (pain) to be filled for one week following D5 and for one week prior to the next tDCS block. 

**Figure 3:**
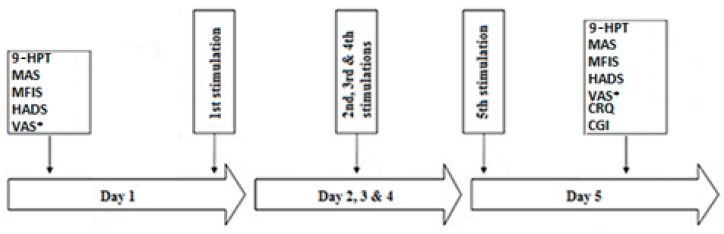
The schematic diagram of the study protocol. 9-HPT: Nine-Hole Peg Test; CGI: Clinical Global Impression; CRQ: Comfort Rating Questionnaire; HADS: Hospital Anxiety and Depression Scale; MAS: Modified Ashworth scale; MFIS: Modified Fatigue Impact Scale; VAS*: Visual Analogue Scale for the pain recorded one week before and after the beginning of each block.

Each patient will be included in the study for 3 months. 2 patients per week will undergo the stimulation block. The study will take place for a total duration of 12 months, and the final data analysis will be performed in less than a year. The schematic diagram of the protocol is illustrated in Figure 3. 

### 2.6. Patient Withdrawal, Risks and Benefits

No available conditions in the study protocol can be expected to trigger patient’s withdrawal. However, any patient can withdraw consent at any point during the study without any consequences. The side effects related to tDCS are minimal to none. Mild skin irritation has been rarely reported and can be avoided by dipping the stimulating electrodes in a saline solution before installation. Protocols of tDCS have been the subject of many studies with no serious side effects described to date. A well-experienced team will be in charge of performing tDCS. This study is designed to improve knowledge regarding tDCS in MS patients with altered dexterity and cannot provide direct or lasting medical benefit, but the obtained results might serve in guiding the therapeutic use of tDCS in this domain.

### 2.7. Data Management

The study records will be kept as confidential as possible. Patients’ information will be carefully collected. What is learnt from the data will be described only in a way that does not identify patients. To protect the patients’ privacy, data will be linked to a secret code. Names will be recorded only on the informed consent forms. The secret codes will be kept in a locked file and can only be accessed by the principal investigator of the study and the authorized personnel. However, study records may be reviewed by the Committee of Human Subjects in Research at the Lebanese American University. The records will be monitored and may be audited without violating confidentiality. The results of this study may be published without breaching patients’ confidentiality at any point.

### 2.8. Safety and Adverse Event Reporting

Safety measures have been well proven by Nitsche et al. In fact, the authors demonstrated that tDCS does not cause heating effects under the electrodes and does not elevate the serum neuron-specific enolase level [30,31,33], a sensitive marker of neuronal damage [66]. It does not result in changes of diffusion-weighted or contrast-enhanced MRI, or of pathological electroencephalographic patterns [33]. These protocols were tested in more than 3000 subjects worldwide with no serious adverse events (SAEs), except for some adverse events (AEs) consisting of slight itching under the electrode, mild redness attributed to neutrally driven vasodilation and seldom-occurring headache, fatigue, and/or nausea [32,35]. tDCS was studied in the context of MS, and its efficacy and safety were reported in the context of neuropathic pain [67,68], tactile sensory deficit [69], and fatigue [40,51,70,71,72,73,74,75], to cite a few. Based on the above, we believe that this study design (5 consecutive daily sessions, 20 min per session, and current intensity of 2 mA, with the proposed circuit setup) is safe and harmless.

The investigators are in charge of recording and reporting all the SAEs that might happen throughout the entire research protocol from the time of obtaining consent and throughout the whole period required to monitor the participants. SAEs will be recorded on a comprehensive form provided for this purpose. This form will be completed, printed, dated, signed, and the principal investigator will be promptly notified. Moreover, regardless of the time of onset following the protocol, all the SAEs suspected to be the result of the research protocol should be reported to the investigator when no other reasonable explanation exists. All the other AEs will be reported only on the medical card of each patient and will include the date of onset, characteristics, intensity, duration, etiologies, taken actions, treatments, and resolutions, if any. There are no specific safety measures related to this research and no important safety data to be collected.

### 2.9. Statistical Analysis

The statistical analysis will be performed using the SPSS software (IBM Corp. Released 2016. IBM SPSS Statistics for Windows, Version 24.0. Armonk, NY: IBM Corp). The primary and secondary outcomes will be analyzed using the same statistical approach. For all variables, the normality of data distribution will be tested using the Kolmogorov–Smirnov test. The homogeneity of variance will be tested using the Levene’s test. Depending on the data distribution, the scores obtained before and after all tDCS conditions will be compared using the repeated measures analysis of variance (ANOVA) or the Friedman test under six conditions: 2 times (before and after tDCS) × 3 stimulation conditions (anodal motor tDCS, cathodal cerebellar tDCS, and sham). When appropriate, the post hoc Dunn’s test will be applied. Two-tailed p-values less than 0.05 will be considered significant. Data will be presented as the mean ± the standard deviation or the median (1st and 3rd quartiles).

## 3. Discussion

The current work presents a design of a randomized controlled trial to assess tDCS effects on manual dexterity in MS patients. Patients will be randomly allocated to receive 3 tDCS blocks each constituted of 5 consecutive daily sessions of anodal motor tDCS, cathodal cerebellar tDCS, and sham over either cortical site. Each session will last 20 min, during which a current of 2mA in intensity will be delivered. This study design (i.e., protocol duration, session duration, and current intensity) was adapted from other studies where tDCS was applied in MS patients in the context of other symptoms [38,40]. So far, no randomized controlled trials have investigated the plausible tDCS effects on hand dexterity in MS patients. The choice of polarity was based on the results obtained following the application of repetitive transcranial magnetic stimulation (rTMS), which is another NIBS technique. High frequency rTMS applied over the motor cortex improved hand dexterity in MS subjects with cerebellar impairment possibly by increasing the motor cortex excitability [76,77]. In another study involving patients with essential tremor, low frequency rTMS applied over the cerebellum was able to improve the amplitude of tremor, a finding that might have occurred due to the decrease in excitability of the cerebellar cortex [78]. Following the same logic, anodal or cathodal tDCS could act by respectively increasing (i.e., anodal tDCS) or decreasing (i.e., cathodal tDCS) the cortical excitability of the stimulated areas [36,37]. This was behind the choice of using anodal tDCS aiming to increase the excitability of M1, and cathodal tDCS aiming to decrease the excitability of the cerebellar cortex in an attempt to release the inhibition exerted by the cerebellum over M1.

It is important to mention the various drawbacks that this study protocol might have. First, stimulation of the cerebellar cortex and/or M1 would also impact the lower limb functions, and evaluation of the balance and lower limbs’ motor power could provide additional information on the utility of such approaches. Secondly, we decided to adopt a previously tested setup that was designed to modulate the function of the entire cerebellar cortex; however, targeting the cerebellar cortex ipsilateral to the most affected site would be more efficient than bilateral cerebellar tDCS. Thirdly, although the assessment of long-term effects could help in the determination of the duration of tDCS effects, we thought of focusing our approach on the short-term effects given the exploratory nature of the study. Evaluation of long-term effects would be included in the upcoming research protocols.

## 4. Conclusions

Compared to rTMS, tDCS is portable and relatively simpler, cheaper, and could be fashioned into patient-administered home therapy [79]. This would make it of greater interest than rTMS if it proves its efficacy in this domain. The results of such a study might be promising for MS patients who suffer from altered manual dexterity and therefore may improve their quality of life. This work might also enhance the current understanding of the nature of cerebellar involvement in this clinical population [80]. Finally, regardless of the results, the study may contribute to develop a field that has not been explored yet.
